# Portrait of a Series of Patients with Pheochromocytoma/Paraganglioma from a Reference Center in Brazil: Relevance of Prior Background Features

**DOI:** 10.1590/S1677-5538.IBJU.2025.0094

**Published:** 2025-07-31

**Authors:** José Viana Lima, Nilza M. Scalissi, Suzan M. Goldman, Claudio E. Kater

**Affiliations:** 1 Faculdade de Medicina da Universidade Federal de São Paulo Departamento de Medicina Divisão de Endocrinologia e Metabolismo São Paulo SP Brasil Divisão de Endocrinologia e Metabolismo, Departamento de Medicina, Faculdade de Medicina da Universidade Federal de São Paulo (EPM/UNIFESP), São Paulo, SP, Brasil; 2 Faculdade de Ciências Médicas da Santa Casa de São Paulo Departamento de Medicina Divisão de Endocrinologia São Paulo SP Brasil Divisão de Endocrinologia, Departamento de Medicina, Faculdade de Ciências Médicas da Santa Casa de São Paulo, São Paulo, SP, Brasil; 3 Fleury Medicina e Saúde Grupo Fleury São Paulo SP Brasil Fleury Medicina e Saúde, Grupo Fleury, São Paulo, SP, Brasil; 4 Faculdade de Medicina da Universidade Federal de São Paulo Departamento de Diagnóstico por Imagem São Paulo SP Brasil Departamento de Diagnóstico por Imagem, Faculdade de Medicina da Universidade Federal de São Paulo (EPM/UNIFESP), São Paulo, SP, Brasil

**Keywords:** Hypertension, Hypotension, Orthostatic, Pheochromocytoma, Metanephrine

## Abstract

**Purpose:**

Pheochromocytomas (Pheo) and paragangliomas (PGL) are catecholamine-secreting tumours, whose functionality is confirmed by elevated plasma (Pl) and/or 24-h urinary (Ur) metanephrines (MN). Relevance of prior background features were reviewed in a large cohort of patients with Pheo and PGL.

**Material and Methods:**

We reviewed clinical, hormonal, and imaging aspects of 116 patients studied prospectively: 93 Pheo; 22 PGL; one Pheo plus PGL.

**Results:**

Twenty-five % PPGL were discovered incidentally. Systemic arterial hypertension (SAH) was present in 81% (43% on stage 3), whereas 9.5% were prehypertensive and 9.5%, normotensive. SAH plus paroxysms occurred in 31 (32.9%) patients, being exclusively sustained in the remaining; 26 (28%) had resistant SAH. Orthostatic hypotension was seen in 65% of patients. Pl/Ur MN and normetanephrine (NMN) were compared to those of a positive (56 functioning PPGL) and a negative control group (654 subjects with normal MN/NMN). Total and fractionated Ur MN were elevated in 94% PPGL patients. Cut-off values of 885 mcg/24-h for Ur MN, and of 1.5 nmol/L for Pl MN identified functioning lesions with 100%/100% sensitivity and 93%/97% specificity, respectively. MRI detected 56% right-side Pheo, 25% on the left, and 19% bilateral; PGL were 56.5% (13/23) retroperitoneal and 43.5%, cervical (10/23). Right-side Pheo were larger (5.8 cm) than left-side ones (3.7 cm), but retroperitoneal (6.5 cm) and neck PGL (6.9 cm) were similar. Tumour size positively correlated with total Ur MN.

**Conclusions:**

in this large cohort of PPGL patients we highlighted relevant aspects of SAH, the frequently overlooked manifestation of orthostatic hypotension, common incidental presentation, significant tumour size/hormonal production.

## INTRODUCTION

Pheochromocytomas (Pheo) and paragangliomas (PGL) are uncommon catecholamine-secreting tumors of the adrenal medulla and extra-adrenal chromaffin tissue, respectively. PGL usually develops from the sympathetic paraganglia of the chest and abdomen. Both are derived from ectodermal neural crest cells and are grouped under the term Pheo/PGL (PPGL) syndrome (1). Recently, the WHO recommended the single name paraganglioma to designate both adrenal and extra-adrenal pheochromocytomas (2, 3).

The incidence of PPGL is between 500 and 1,600 cases per year, with an equal sex distribution and the highest frequency in the 4th and 5th decades of life. PPGL has a prevalence of 0.1 to 0.6% among the hypertensive population (4–6).

PPGL manifests clinically as sustained arterial hypertension that is usually accompanied by paroxysms (headache, palpitation, and sweating) and refractoriness to antihypertensive medications. Silent PPGL may be found as adrenal incidentalomas. With more genetic discoveries and detection tools, hereditary syndromes will become more common, whereas sporadic disease was the rule in the past (6–10).

Laboratory confirmation is based on elevated levels of plasma and/or urinary fractionated metanephrines. Plasma and urinary catecholamines can also be used, but are less sensitive, as is urinary vanillylmandelic acid (VMA), which was used in the past (11–16). The imaging location of unilateral or bilateral Pheo and cervical (head and neck), thoracic and pelvic PGL can be found by both CT and MRI, which may be functionally complemented by scintigraphy with ^131^I-mIBG (meta-iodobenzylguanidine) and ^68^Ga-DOTATATE (DOTA-octreotate) (6, 8, 13, 17–20).

This article will focus on the major clinical, hormonal, and imaging aspects of a large series of PPGL patients studied in a single endocrine reference center in São Paulo, state of São Paulo, Brazil.

## MATERIAL AND METHODS

### Study population

From 2001 to 2019, we prospectively studied 137 patients ranging in age from 12 to 82 years in whom a diagnosis of Pheo and/or PGL was clinically considered and later confirmed by (i) hormonal assessment, (ii) specific imaging procedures, (iii) genetic evaluation for germline variants, (iv) surgery, and (v) pathological examination. Twenty-one patients were later excluded: 18 declined further participation, and three were under 14 years, the limit age per our protocol. The remaining 116 were 75 females (64.7%) and 41 males, with a median age of 45 years (ranging from 14 to 79 years). All patients were referred to the Adrenal and Hypertension Outpatient Clinic of the Division of Endocrinology and Metabolism, Department of Medicine at EPM/UNIFESP for 1) evaluation of a possible secondary form of hypertension; 2) the presence of a suspicious adrenal or extra-adrenal mass; and 3) the existence of first-degree relatives with PPGL.

### Control groups

To analyse and compare values of plasma and urinary metanephrines, we used two control groups, both extracted from an uncharacterized database registry of the Fleury Group, São Paulo, Brazil, after formal authorization: one consisted of data from patients with elevated plasma and/or urinary metanephrines in whom a diagnosis of PPGL was unequivocally established (positive control group); the other comprised data from a large cohort of subjects who had been evaluated for different disorders and included a panel of plasma and/or urinary metanephrines that were all normal. None of those patients were diagnosed with a PPGL at that time (negative control group); non-functioning PPGL could not be excluded.

Positive controls included 56 subjects: 33 females (58.9%) and 23 males ranging in age from 26 to 82 years (median of 54). Fifty of them had Pheo and six had PGL. They were all "functioning" PPGL (hormone-secreting) by definition.

The negative control were 654 subjects: 412 females (63%) and 242 males ranging in age from 16 to 82 years (median of 51). All had plasma and urinary metanephrines within the Fleury laboratory's reference ranges. "Nonfunctioning" PPGL could not be excluded in them.

All 116 patients from this study cohort (and/or their parents or liable) signed a written informed consent form for all investigational procedures and therapeutic decisions, which were previously approved by the Committee of Ethics in Clinical Research of the Institution. Patients from the positive and negative control groups were individually investigated by their respective physicians. Authorization to use the biochemical data employed in this manuscript, plus sex, age and information that led to the final diagnosis, was provided by the responsible Board of Directors of Fleury Group without personal patient identification.

## STUDY DESIGN

The following data were obtained from all patients at the time of admission: sex and age, height and weight (and body mass index, BMI), systolic and diastolic blood pressures and heart rate (HR), specific manifestations of the disease (such as sustained hypertension and/or paroxysms, tachyarrhythmia, orthostatic hypotension, cardiogenic shock), impaired glucose tolerance (prediabetes or diabetes mellitus), presence of comorbidities, family history of PPGL and specific conditions suggesting the presence of germline pathogenic variants (PVs, see below), and current or past use of antihypertensive medications. We used the 2020 Brazilian Guidelines of Hypertension to grade the patient's arterial hypertension (21).

Conditions suggesting the presence of germline PVs were as presented elsewhere (22–24).

## LABORATORY EVALUATION

### Urinary and blood collections

All patients clinically suspected underwent hormonal evaluation directed towards the hormonal detection of PPGL. Up to 2014, we employed 24-h urinary collections to measure 1) catecholamine excretion: norepinephrine [NE], epinephrine [E], and dopamine [Dp], 2) fractionated metanephrines: normetanephrine [NMN], metanephrine [MN], and 3-methoxy-tyramine [3MT], and 3) vanillylmandelic acid (VMA). In addition, we simultaneously withdrew blood to measure plasma catecholamines (NE, E, and Dp). From 2014 on, as per the Endocrine Society's PPGL Guideline (8), we started measuring plasma NMN and MN in addition to 24-h urinary metanephrines and discontinued urinary and plasma catecholamines.

In several patients, we also measured plasma chromogranin A by immunoassay or targeted proteomics, and when indicated, we performed a clonidine test (0.3 mg PO with blood collected before and after 3h) and measured plasma catecholamines or metanephrines.

### Hormonal measurements

We measured plasma catecholamines, 24 h urinary catecholamines and metanephrines, and VMA by high-performance liquid chromatography (HPLC) using the Chromsystem Commercial Kit. [Chromsystems Instruments & Chemicals, GmbH].

Plasma metanephrines were determined by HPLC coupled to tandem mass spectrometry (LC-MS/MS) through an in-house method developed and validated at the Fleury Group in 2010 [unpublished], in consonance with other protocol (25).

### Radiological/Imaging evaluation

We performed the following imaging procedures, as indicated and available: 1) abdominal (adrenal) and pelvic computerized tomography (CT) and/or magnetic resonance imaging (MRI); 2) cervical and/or chest MRI for patients with a suspected head/neck or thoracic tumor; 3) ^131^I-mIBG full body scintigraphy; and 4) ^18^FDG and/or ^68^Ga-DOTATATE or DOTATOC PET-CT.

We analysed the following CT and MRI data: topography, size, and characteristics of the lesion.

We performed whole-body ^131^I-mIBG scintigraphy if the image was uncertain, dubious, or suggestive of the presence of metastases (on clinical or hormonal grounds) and in cases of confirmed metastasis to evaluate the possibility of radiopharmaceutical therapy.

We analysed the following data: moderate/strong uptake in adrenal and/or extra-adrenal topographies and uptake in target organs of metastasis (bones, liver, lungs, and lymph nodes).

Not all biochemical and imaging diagnostic procedures were necessary for each patient. We requested specific hormonal and topographic tests only for diagnostic purposes, mainly 24-h urinary NMN/MN and adrenal MRI or CT and/or ^131^I-mIBG and ^18^FDG PET-CT, whenever necessary and available.

### Genomic evaluation

Genetic analysis was performed in 115 patients to determine the familial (hereditary) or sporadic nature of their respective diseases using a panel that had the following 23 PPGL-related genes: ATM, ATR, CDKN2A, EGLN1, FH, HRAS, KIF1B, KMT2D, MAX, MDH2, MERTK, MET, NF-1, RET, SDHA, SDHAF2, SDHB, SDHC, SDHD, TMEM127, TP53, VHL, and PIK3CA. The results of the genomic studies are reported elsewhere (22).

## Statistical Analysis

All patient variables were deposited in a computer program database (Microsoft Excel®) and updated at each follow-up visit. For statistical purposes, all nondetectable values were arbitrarily considered equal to the limit of sensitivity for the assay divided by the square root of 2 (26). We performed parametric and nonparametric statistical tests according to the nature of the variable, which were tested for normality by the Kolmogorov–Smirnov test. Descriptive analysis included absolute (n) and relative (%) frequencies and bar graphs of qualitative variables and summary measures (mean, standard deviation, median, minimum, and maximum). Inferential analysis included the association test using Chi-square or Fisher's exact test and the logistic regression-stepwise forward method and ROC curve analysis to evaluate possible cutoff points for quantitative variables, complemented by calculation of sensitivity and specificity. Cut-off points were established by ROC curve analysis as recommended (27). P<0.05 was significant.

## RESULTS

Among the 116 PPGL patients in the present series, 102 were index cases (probands), and 14 were relatives from 8 families.

### Anthropometry and clinical presentation

**Figure 1:**
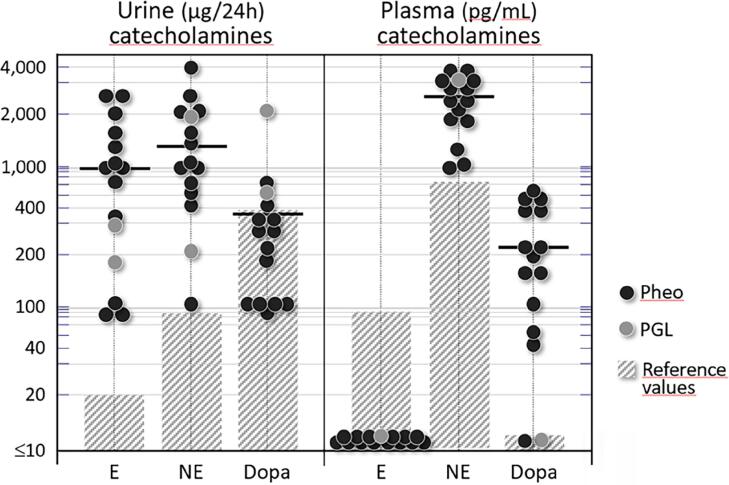
Individual values of fractionated plasma and 24-h urinary catecholamines (epinephrine, norepinephrine, and dopamine) from 15 patients with PPGL. Shaded areas represent reference laboratory values. Note the semilogarithmic scale; horizontal bars denote means.

Table - 1 depicts anthropometric and clinical features of the 116 PPGL patients, separated into Pheo (n= 94) and PGL (n= 22). Among the 94 Pheo patients, one patient also had a neck PGL (thus, in Table-2 and Figure-1, total PGL are 23). The sex ratio was approximately 2 F:1 M. PGL patients were younger (35.5 vs. 45 years, p<0.01) and comprised significantly more subjects ≤20 years of age than Pheo (18.2% vs. 6.4%; p<0.01). Median BMI and heart rate were similar between groups.

**Table 1:** Anthropometric and clinical data from all 116 PPGL patients, divided into pheochromocytoma (Pheo) and paraganglioma (PGL). Data are mean ±SEM, median and range, and absolute and percent values.

		Pheochromocytoma	Paraganglioma	All PPGL	p
N=		94*	22	116	
F (%) / M		60 (64%) / 34	15 (68%) / 7	75 (65%) / 41	NS
Age, y (range)		45 (14-72)	35.5 (14-79)	45 (14-79)	<0.001
Age ≤ 20y (%)		6 (6.4%)	5 (22.7%)	11 (9.5%)	<0.001
BMI (kg/m^2^)		25.0 ± 0.4	25.5 ± 1.0	25.1 ± 0.4	NS
median [range]		24.5 [17.7-42.0]	25.3 [16.5-36.6]	24.6 [16.5-42.0]	
Functioning PPGL		93 (98.9%)	13 (59.1%)	106 (91.3%)	<0.05
Metastatic		4 (4.3%)	7 (31.8%)	11 (9.5%)	<0.001

Heart rate (bpm)		85.0 ± 1.0	86.7 ± 2.8	85.3 ± 1.0	NS
median [range]		88 [64-100]	88 [68-120]	88 [64-120]	

Normal BP		9 (9.6%)	2 (9.1%)	11 (9.5%)	
Pre-hypertension		7 (7.5%)	4 (18.2%)	11 (9.5%)	NS
Hypertension		78 (83%)	16 (72.7%)φ	94 (81%)	
Paroxysms only		6 (6.4%)	1 (4.6%)	7 (6.0%)	

**Post. hypotension**		65 (69.1%)	10 (45.5%)	75 (64.7%)	<0.05
	Witht Htn (%)	59 (90.8%)	10 (100%)	69 (92%)	
	Without Htn (%)	6 (9.2%)	0 (0%)	6 (8%)	

**Only hypertensives:**					
	− Stage 1 Htn	25 (32.1%)	3 (18.8%)	28 (29.8%)	
	− Stage 2 Htn	21 (26.9%)	4 (25%)	25 (26.6%)	NS
	− Stage 3 Htn	32 (41%)	9 (56.3%)	41 (43.6%)	

SBP (mmHg)		168.3 ± 2.9	173.4 ± 8.0	169.1 ± 2.8	
median [range]		165 [135-270]	170 [130-250]	170 [130-270]	
DBP (mmHg)		99.6 ± 1.7	110.0 ± 5.5	101.4 ± 1.7	
median [range]		95 [70-150]	100 [80-150]	100 [70-150]	

Sustained BP only		31 (39.7%)	8 (50.0%)	39 (41.5%)	
Sustained + Paroxysms		47 (60.3%)	8 (50.0%)	55 (58.5%)	NS
Difficult control		20 (25.6%)	6 (37.5%)	26 (27.7%)	

* includes one pt. with both a right pheo and a neck PGL;

ϕ hypertensive neck PGLs were possibly essential.

Patients were referred for investigation mainly owing to systemic arterial hypertension (SAH; 81%) (see below), but also due to a previous positive genetic screening (26.7%), the presence of an adrenal incidentaloma (23.4%), and other reasons (11.2%).

Virtually all Pheo (98.9%) (including the one combined with a neck PGL) and 59.1% of PGL were functioning lesions, in the sense that they produced excessive catecholamines that resulted in clinical manifestations. At presentation, 64.7% of the patients had two clinical manifestations, 25% had only one, and 13.8% had three or more. The most common clinical manifestation was SAH in its various forms.

### Blood pressure

**Figure 2:**
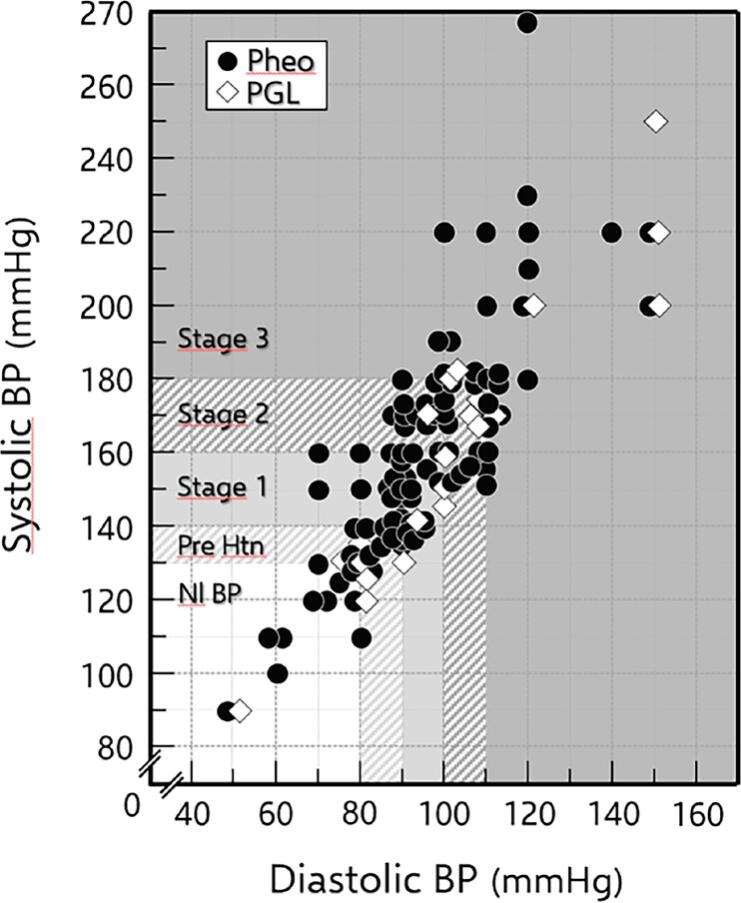
Individual paired values of systolic and diastolic blood pressure from the 116 PPGL patients studied. Background shaded grades define areas of normal blood pressure (Nl BP), prehypertension (pre-Htn), and stages 1 to 3 hypertension. Solid circles denote pheochromocytomas; open lozenges denote PGL.

Table-1 and Figure-2 show the results related to hypertension. SAH was present in 94 of the 116 PPGL patients (81%), whereas 11 (9.5%) were prehypertensive and 11 (9.5%) had normal blood pressure. Among the 94 hypertensive patients, 28 (29.8%) were in stage 1, 25 (26.6%) were in stage 2, and 41 (43.6%) were in stage 3. Stage 3 SAH prevailed over stages 1 and 2 in the whole PPGL group, especially among the PGL patients. Overall, systolic and diastolic blood pressure (BP) were higher in hypertensive PGL than in Pheo, albeit not significantly.

High BP was found alone in 29 (37.2%) hypertensive PPGL patients (29 Pheo) and was accompanied by paroxysms in the remaining 31 (32.9%) (29 Pheo). Hypertension that was difficult to control (resistant SAH) was present in 26 (27.7%) PPGL patients (20 Pheo) presented paroxysmal and combined hypertension. Seventy-five (64.7%) patients (65 Pheo) had orthostatic hypotension, six of whom (8%, all Pheo) did not have hypertension of any form (all genetic [cluster 2], hormonally secretory, and generally smaller in size).

The estimated median time for presentation of SAH (with or without paroxysms) was 24 month (ranging from 1 to 480 months).

### PPGL-associated comorbidities

Impaired glucose tolerance was the most frequently associated comorbidity, present in 18.1% of cases. These other comorbidities were mostly associated with genetic disease: medullary thyroid carcinoma (15%), type 1 neurofibromatosis (7.8%), primary hyperparathyroidism (4.3%), retinal angioma and central nervous system (CNS) hemangioblastomas (3.4%), and clear cell renal carcinoma (3.4%).

### Metastases

Recurrence of PPGL and metastasis occurred in 11 (9.5%) patients (Table-1): seven PGL (six with germline PVs in the SDHB gene and one in the VHL gene) and four Pheo (one with a PV in the NF1 gene). Metastases involved lymph nodes in all, blood vessels in eight, bone (spine and pelvis) and liver in five each, lungs in three, and CNS (head and neck PGL) in two.

Two patients relapsed after 12 months, five after 36 months, and one each after 5, 28, and 33 years. The last two patients have already been discharged from their respective medical services after 10 years of uncomplicated follow-up. Nevertheless, paroxysmal hypertension and a syndrome of weight loss/sarcopenia manifested in them after another 18 and 23 years, respectively.

### Hormonal presentation

#### Urinary and plasma metanephrines

With the large negative control population (n= 654) as reference, we defined our own reference ranges for total and fractionated 24-h urinary and plasma MN as follows (results in mean ± SE, and [range]): 24-h urinary NMN: 214.8 ±4.0 mcg/24 h [15-784]; MN: 81.9 ±1.6 mcg/24 h [3.5-326], and total metanephrines: 296.7 ±4.7 mcg/24 h [33-878]. Plasma NMN: 0.45 ±0.01 nmol/L [0.14-1.0] and MN: 0.18 ±0.01 nmol/L [0.14-0.6), and total metanephrines: 0.63 ±0.01 nmol/L [0.28-1.5].

**Figure 3:**
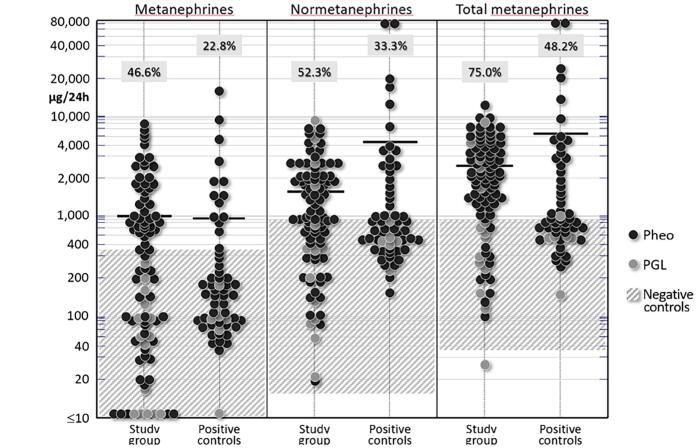
Individual values of total and fractionated 24-h urinary metanephrines from 89 patients with PPGL and 56 positive controls (see text for explanation). Shaded areas represent the normal ranges (minimum and maximum values from the negative control group). Note the semilogarithmic scale; horizontal bars denote means. Boxed numbers atop each series represent the percentage of values above the upper limit of normal (ULN).

As shown in Table-3, 88 PPGL patients from the present study and, especially, 56 from the positive control group had significantly higher levels of NMN, MN, and total MN than the negative controls. The individual values of 24-h urinary MN from these subjects are shown in Figure-3.

**Table 3:** Urine and plasma total and fractionated metanephrines from 88 PPGL study patients and the positive and negative control groups. Their laboratory upper reference values are written (see text for explanation). Data are mean ± SEM, median and range, or n (%).

	Pheochromocytoma	Paraganglioma	All PPGL	Positive Controls (Pheo)	Positive Controls (PGL)	Positive Controls (All PPGL)	Negative Controls	Reference
24h-Urine data (μg/24h)	**N= 71**	**N= 17**	**N= 88**	**N= 50**	**N= 6**	**N= 56**	**N= 654**	
**NMN** (mean ± SE)	1,354 ± 158	1,748 ± 554	1,430 ± 165	4,860 ± 2,026	437 ± 65	4,387 ± 1,817	214.8 ± 4.0	< 800
median [range]	971 [21-5,800]	600 [48-7,543]	892 [21-7,543]	697 [150-72,768]	425 [217-716]	639 [150-72,768]	201 [15-784]	
**MN** (mean ± SE)	1,076 ± 168	112 ± 47	890 ± 142	911 ± 350	82 ± 22	822 ± 315	81.9 ± 1.6	< 400
median [range]	580 [3.5-6,572]	50 [3.5-800]	277 [3.5-6,572]	147 [37-15,453]	85 [4-170]	145 [4-15,453]	76 [3.5-326]	
**Total MN** (mean ± SE)	2,430 ± 239	1,860 ± 540	2,320 ± 219	5,771 ± 2,048	519 ± 87	5,208 ± 1,840	296.7 ± 4.7	< 1.200
median [range]	1,950 [28-10,300]	675 [112-7,547]	1,882 [28-10,300]	859 [258-72,835]	497 [221-886]	822 [221-72,835]	286 [33-878]	
**Plasma data (nmol/L)**	**N= 14**	**N= 6**	**N= 20**	**N= 50**	**N= 6**	**N= 56**	**N= 654**	
**NMN** (mean ± SE)	29.9 ± 7.3	7.1 ± 5.6	23.0 ± 5.8	19.1 ± 10.4	1.5 ± 0.2	17.2 ± 9.3	0.45 ± 0.01	< 0.9
median [range]	21.9 [0.6-80.2]	1.6 [0.6-35]	8.6 [0.6-80.2]	2.3 [1-502]	1.5 [1-2.3]	2 [1-502]	0.4 [0.14-1.0]	
**MN** (mean ± SE)	11.4 ± 4.5	0.5 ± 0.1	9.0 ± 3.4	1.5 ± 0.5	0.2 ± 0.0	1.4 ± 0.4	0.18 ± 0.001	< 0.5
median [range]	2.2 [0.2-45.0]	0.4 [0.2-0.9]	1.0 [0.2-45.0]	0.3 [0.1-17.2]	0.2 [0.1-0.3]	0.3 [0.1-17.2]	0.14 [0.14-0.6]	

MN = metanephrines;

NMN = normetanephrines

A cut-off value of 885 mcg/24 h for total urinary MN, determined by ROC curve analysis, had 100% sensitivity and 92.5% specificity at separating functioning from nonfunctioning PPGL (study cases and positive controls vs. negative controls). In fact, 67.4% of the 24-h urinary total MN values were above 885 mcg/24 h in the subgroup of functioning PPGL from this study and the positive control groups, compared to none in the negative controls (Figure-3).

Table-3 also shows that plasma metanephrines (NMN and MN) from the study group (n= 20) and the positive control group (n= 56) were significantly higher than those from the negative control group (p<0.01). A cut-off value of 1.5 nmol/L for total plasma MN determined by ROC curve analysis had 100% sensitivity and 97.3% specificity to separate functioning from nonfunctioning PPGL lesions (study cases and positive controls vs. negative controls).

### Urinary and plasma catecholamines

The following results were obtained for 24-h urinary catecholamines from 13 patients with Pheo and two with PGL: epinephrine: 906 ± 216 mcg/24 h (800; range: 70-2,500); norepinephrine: 1,275 ±263 mcg/24 h (900; range: 100-3,900); and dopamine: 360 ±123 mcg/24 h (250; range: 70-2,000). Figure-1 depicts all the individual values.

None of the PPGL patients had detectable values for plasma epinephrine, whereas plasma norepinephrine was 2,373 ±221 pg/mL (2,500; range: 900-3,500) and dopamine was 215 ±42 (180; range: 28-500). Individual values are shown in Figure-1.

### Clonidine test and chromogranin A

Clonidine tests were performed in seven PPGL patients (six Pheo) and were positive in all.

Plasma chromogranin A was above 800 ng/mL (reference values <93 ng/mL) in three patients with metastatic PPGL.

### Topographic distribution and tumor size

Most PPGL patients (93.9%) were initially imaged by MRI instead of CT (given the ease of performing it in our service). Imaging features from all patients are shown in Table-3: 53 Pheo (56.4%) were located on the right side, 23 (24.5%) on the left, and 18 (19.2%) were bilateral. Among the 23 PGL, 13 (56.5%) were retroperitoneal and 10 (43.5%) were cervical.

**Figure 4:**
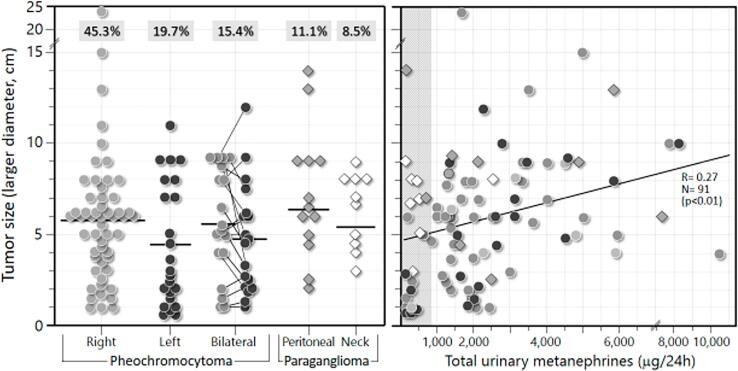
Individual location and size of lesions from 116 PPGL patients. Size is the largest diameter (in cm) obtained by CT or MRI. A) Horizontal bars denote means. Boxed numbers atop each series represent percentages of total PPGL. B) Correlation between lesion size and 24-h urinary excretion of total metanephrines from 89 patients with PPGL. Represented are either unilateral pheochromocytomas or the larger lesion of the bilateral ones.

Table-2 also shows tumor sizes (larger diameter). Note that the Pheo located on the right side were significantly larger than those on the left (including the bilateral ones) (median of 5.8 vs. 3.7 cm, respectively) (p<0.01). Retroperitoneal and neck PGL had similar sizes (medians of 6.5 and 6.9 cm, respectively). The individual sizes of all PPGL lesions are depicted in Figure-4. Note that 70% of all 117 PPGL lesions were larger than 3 cm in diameter (and 61% were larger than 4 cm).

**Table 2:** Imaging data from all 117 PPGL lesions (116 patients*), divided into pheochromocytoma (Pheo) and paraganglioma (PGL). Data are mean ± SEM, median and range, or n (%).

		Pheochromocytoma	Paraganglioma	All PPGL lesions
N		94*	23*	117
**Imaging data**				
	MRI / CT	87 / 10	23 / 1	110 / 11
	Incidentaloma	22 / 94 (23.4%)	0 / 23	22 / 117 (18.8%)
	Unilateral Pheo (R/L)	53 (56.4%) / 23 (24.5%)	----	76 (65.5%)
	Bilateral	18 (19.2%)	----	18 (19.2%)
Size^#^ R/L (cm)		5.8 ± 0.4 / 4.5 ± 0.5	----	5.3 ± 3.3
median [range]		5.8 [1-24] / 3.7 [0.6-12]	----	5.0 [6-24]
Retroper/Neck*		----	13 (56.5%) / 10 (43.5%)	23 (19.6%)
Size Retr/Neck (cm)		----	7.2 ± 1.0 / 6.3 ± 0.7	6.8 ± 6.2
median [range]		----	6.5 [2.2-14] / 6.9 [3-9]	6.8 [2.2-14]
**Functional imaging**				
	^131^I-mIBG	77	14	91
	Unilateral Pheo (R/L)	40 (52.0%) / 21 (27.3%)	----	61 (67.0%)
	Bilateral	16 (20.7%)	----	16 (17.6%)
	Retroper/Neck*	----	9 (64.3%) / 5 (35.7%)	14 (15.4%)

* = includes one pt. with both a right pheo and a neck PGL;

# = includes bilateral lesions (all right side = 71; all left side= 41).

High signal intensity on the T2-weighted sequence was observed in 108 of the 109 patients (99.1%) who underwent MRI. Among the 11 PPGL patients who underwent CT imaging, all typically had pre-contrast attenuation values above 10 HU, above 20 HU in the portal phase, and a contrast washout <60%.

#### ^131^I-mIBG whole-body scintigraphy

As shown in Table-2, 91 PPGL patients underwent ^131^I-mIBG scintigraphy: 77 in the Pheo group (40 right-side, 16 bilateral) and 14 in the PGL group (nine retroperitoneal).

Fifty-seven PPGL patients (52.3%) underwent both MRI and ^131^I-mIBG scintigraphy: 29 (50.9%) had right-side and 17 (29.8%) left-side Pheo (including one with a neck PGL), six (10.5%) had retroperitoneal and three (5.3%) neck PGL, and two (3.5%) had bilateral Pheo. The concordance between both procedures was 98.2%. The only discordance occurred in the patient with adrenal Pheo and neck PGL, in whom uptake was observed only in the left adrenal Pheo. ^131^I-mIBG scintigraphy was done in 30 (63.8%) of the 47 patients carrying PVs: uptake was observed in 29 of them (22).

In eight PPGL patients (two Pheo with an SDHB gene PV), metastases were present or developed during follow-up. Lesions were identified in the spine and pelvis by ^68^Ga-DOTATATE PET-CT.

#### Metanephrines and tumor size

A significant positive correlation was observed between tumor size (larger diameter, in cm) and total urinary MN (in mcg/24 h) for 91 functioning PPGL (74 Pheo and 17 PGL): r= 0.27; p<0.01 (Figure-4). Nonfunctioning PPGL, especially head and neck PGL, presented 24-h urinary MN within the normal range.

## DISCUSSION

PPGL is a rare neuroendocrine tumor that is potentially metastatic and fatal if this diagnosis is not promptly suspected and treatment is not started soon. Its incidence has been increasing recently due to improvements in the measurement of catecholamine metabolites and a greater demand for imaging tests. Unfortunately, there is still a significant diagnostic delay, some diagnosis only coming postmortem (2, 3).

This diagnostic delay was confirmed in our population sample: most patients took two or more years to seek medical assistance, and one of them took almost 40 years to be diagnosed. Hence, major complications related to hypertension are to be expected.

PPGL is one of the main diagnostic possibilities in the investigation of secondary and/or resistant forms of SAH, regardless of the presence of paroxysms. Clinical scores are available to help define an individual's risk of having PPGL. Features such as hyperhidrosis, palpitations, pallor, tremors, and nausea are 30-90% more prevalent in the PPGL population than in other hypertensive patients. These characteristics plus a heart rate above 85 bpm and BMI <25 kg/m2 guarantee scores close to the maximum of 7 points, a score that makes PPGL 5.8 times more likely than lower scores do (14).

The main manifestation in our patients that led to the suspicion of PPGL was sustained SAH. Other common ones were paroxysms, genetic screening, difficult-to-control SAH and adrenal incidentalomas, consistent with the medical literature (7, 13, 19). When first investigated, most patients were already taking two or more classes of antihypertensive drugs. Orthostatic hypotension was a mostly relevant finding, often associated with SAH but also found in normotensive patients who were initially referred for genetic screening or evaluation of adrenal incidentaloma. This finding is important in the initial evaluation, as it is often present whether the individual has SAH or not.

Normotensive and prehypertensive patients (almost 20% of our sample) were referred for evaluation of PPGL due to a positive family history or presence of adrenal incidentaloma (22%), as has been reported (19, 22–24, 28, 29, 31, 32). These data also help to dismiss the outdated "rule of 10%" for PPGL.

Investigation of PPGL is recommended for recent-onset diabetes mellitus in a young, lean, hypertensive patient since catecholamine excess favours hepatic gluconeogenesis and less degranulation of insulin by pancreatic beta cells (13). Eighteen percent of our patients had impaired glucose tolerance, raising the question whether diagnosis and treatment of PPGL could change some subjects’ DM history, even leading to a "cure".

The landscape of PPGL germline gene variants from 115 of our patients had many pathogenic and likely pathogenic variants, most often in the RET gene, followed by the VHL and SDHB genes (22, 34, 35).

Total plasma MN are considered the gold standard for the diagnosis of functioning PPGL, with slight superiority over 24-h total urinary MN. In the past, the combination of urinary MN with plasma/urinary catecholamines was recommended because of its lowest false negative rate; however, plasma MN alone is diagnostically better than any of the combined tests. Negative plasma MN virtually excludes functional PPGL, but in its absence, 24-h urinary MN can replace them. Thus, the current laboratory diagnosis of PPGL must include plasma and/or 24-h urinary MN (6-9, 13, 15, 30, 33).

Both our PPGL study patients and the positive control group presented very high values of 24-h urinary and plasma MN compared to our large reference negative control group, even when nonfunctioning PPGL (especially those located in the neck) were included. The cut-off value of 885 mcg/24 h had a sensitivity and specificity of 100% and 92.5%, respectively, for total urinary MN. Thus, 24-h urinary MN alone is likely a sensitive and good marker of PPGL functionality, useful for screening patients for the disease.

**Figure 5:**
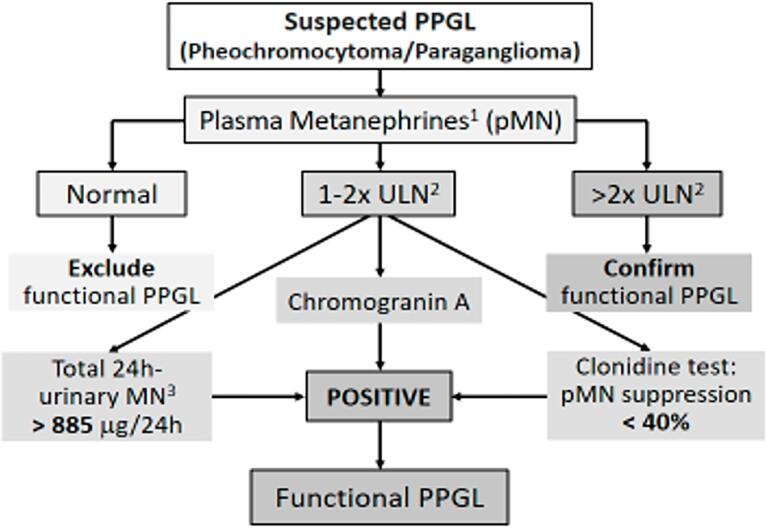
Suggested algorithm for laboratory investigation of functioning pheochromocytoma/paraganglioma (PPGL). 1 Measured by HPLC coupled with tandem mass spectrometry (LC-MS/MS); 2 ULN= upper limit of normal; 3 Measured by HPLC or LC-MS/MS.

Plasma MN values twice above the upper limit of normal are strongly suggestive of PPGL, whereas values between 1X and 2X are considered suspect, requiring diagnostic complementation with 24-h urinary MN and/or chromogranin A measurement and/or a suppression test with clonidine. Based on our findings and others, we suggest a simple and direct algorithm (Figure-5) to diagnose functioning PPGL. The measurement of 3-methoxy-tyramine can help in the diagnosis of PPGL, especially in dopamine-producing, metastatic, and neck PGL (13).

The topographic diagnosis of PPGL should initially be made with CT of the adrenals and pelvis, whereas MRI would be reserved for specific cases such as carriers of PV in PPGL-related genes, intracardiac and head/neck PGL, pregnant women, children, tumor recurrence and metastatic PPGL. Both imaging procedures have good sensitivity and specificity for the diagnosis of PPGL. The characteristics of PPGL on CT are attenuation values in the pre-contrast phase greater than 10 HU, usually above 20, with a contrast washout <60% and high signal intensity ("light-bulb" bright) on the T2-sequence and out-of-phase sequence of MRI, with no loss of signal (8, 9, 13).

The ready availability of MRI over CT in our group prompted us to study 109 of our 116 patients with MRI at first, which was able to identify the high signal intensity on T2 in 108 of them; seven of the patients who underwent adrenal/pelvis CT had characteristics of a nonadenomatous lesion. The tumor diameter in our patients was mostly in the range of 5 to 6 cm. We identified three micropheochromocytomas (the smallest measuring 3 mm in diameter), none of hereditary origin, whose images disclosed only adrenal thickening, but their adrenergic clinical picture was exuberant. Increased hormonal levels were detected during adrenergic episodes. The largest Pheo in our series was 24 cm in diameter and was initially suspected to be a primary adrenal carcinoma.

We found a significant positive correlation between tumor size and total 24-h urinary MN. Functioning PPGL with a diameter greater than 5 cm tend to produce total urinary MN above 1,000 mcg/24 h.

Whole-body scintigraphy with ^131^I-mIBG was employed in 57 of our patients for the following reasons: to search for PGL foci (mainly in hereditary cases) and to investigate metastases, dubious MRI or CT images and the possibility of treatment with radiopharmaceuticals. In all, scintigraphy had excellent agreement with MRI, in addition to accurately mapping hereditary cases and metastases. One patient with a PV in the NF1 gene underwent an exam that was able to identify metastases following tumor recurrence, which made treatment with ^131^I-mIBG possible.

In advanced and aggressive metastatic PPGL cases and in those with PV in the SDHB gene, ^131^I-mIBG scintigraphy was not able to identify all foci of metastasis, unlike ^68^Ga-DOTATATE PET-CT and ^18^FDG PET-CT. Therefore, in these situations, we suggest initially performing ^68^Ga-DOTATATE PET-CT and later performing ^18^FDG PET-CT.

In summary, we present the clinical, hormonal, and imaging picture of a representative cohort of PPGL patients studied in a single reference center in São Paulo, Brazil, whose data mostly agree with other series reported worldwide. Of note, nearly 25% of the Pheo were discovered incidentally. Sustained SAH was present in more than 80% of cases (especially PGL patients), mostly severe and treatment-resistant and typically accompanied by paroxysms. Two-thirds of patients had orthostatic hypotension, an important but generally overlooked diagnostic clue. Diagnostic confirmation of a functioning PPGL strongly relies on the finding of elevated metanephrines (plasma and/or 24-urinary), complemented by chromogranin A and/or a clonidine test, as necessary. MRI was particularly valuable to identify PPGL and to investigate patients with germline variants: the right adrenal gland was more frequently affected than the left, and bilateral masses were present in 20% of cases. The average mass diameter was 5-6 cm, and the larger the mass, the more marked its total urinary metanephrine excretion. ^131^I-mIBG was virtually 100% concordant with MRI. Although uncommon, the diagnosis of PPGL should be considered in young, lean, treatment-resistant hypertensive patients with an impaired glucose tolerance or new-onset diabetes mellitus.
